# APO-CViT: A Non-Destructive Estrus Detection Method for Breeding Pigs Based on Multimodal Feature Fusion

**DOI:** 10.3390/ani15071067

**Published:** 2025-04-07

**Authors:** Jinghan Cai, Wenzheng Liu, Tonghai Liu, Fanzhen Wang, Zhihan Li, Xue Wang, Hua Li

**Affiliations:** 1 College of Computer and Information Engineering, Tianjin Agricultural University, No.22 Jinjing Road, Xiqing District, Tianjin 300392, China; q1463747994@163.com (J.C.); wenzheng_0307@163.com (W.L.); wangfanzhen@tjau.edu.cn (F.W.); zhihan_li@126.com (Z.L.); wangxue01160@163.com (X.W.); hua_l@163.com (H.L.); 2 College of Engineering and Technology, Tianjin Agricultural University, No.22 Jinjing Road, Xiqing District, Tianjin 300392, China

**Keywords:** multimodal, feature extraction, estrus detection, non-destructive, feature fusion

## Abstract

Detecting estrus in sows is important for improving pig reproductive performance and pig farm production efficiency levels. Traditional estrus detection methods are highly subjective and inaccurate, making it difficult to meet the demands of modern farming. This research developed a multimodal feature fusion method that combines audio and thermal infrared image data to enhance the accuracy and robustness of estrus monitoring in breeding pigs. We designed the Adaptive-PIG-OESTUS-CNN-ViT model, which uses thermal infrared images and audio as inputs for a network model. By integrating the Vision Transformer and convolutional neural networks, the model extracted and fused features from multimodal data. An adaptive cross-attention mechanism was employed to automatically learn feature vectors representing the combined thermal infrared and audio data, which were then fed into an improved DenseNet network to identify estrus and non-estrus states in breeding pigs. The model achieved an accuracy of 98.92%, a recall rate of 95.83%, and an F1-score of 97.35%, effectively performing non-destructive estrus detection in breeding pigs. Compared with traditional estrus detection methods, this approach more accurately integrated data from different modalities to distinguish the estrus state of breeding pigs, providing an efficient, objective, and non-destructive means for sow estrus detection.

## Simple Summary

Detecting estrus in sows is vital for pig farming. Traditional methods are subjective and inaccurate. This research developed a multimodal feature fusion method using audio and thermal infrared image data. The Adaptive-PIG-OESTUS-CNN-ViT model achieved high accuracy, providing an efficient, objective, and non-destructive estrus detection method.

## 1. Introduction

In 2023, China’s pig production capacity continued to grow, slaughtering 726.62 million pigs, an increase of 3.81% year-on-year, and pork production reached 57.94 million tons, up 4.6% from 2022 [1]. The global pork output grew by 0.85% year-on-year, totaling 115.5 million tons. Productivity per sow per year (PSY), defined as the number of weaned piglets per sow annually, is a core indicator of sow reproductive efficiency and the technical level of a pig farm. In 2014, China’s PSY was only 17.14, far below levels in developed pig-raising countries. For comparison, Denmark, Ireland, Germany, and the USA had PSYs of 30, 27.8, 27.5, and 26, respectively, in 2014 [2]. In Chinese pig production, 10–30% of replacement gilts exhibit silent estrus that goes undetected due to the lack of visible signs. This delays breeding and negatively affects PSY [3]. The second phase of China’s Pig Genetic Improvement Plan aims to achieve a PSY of over 32 for lean-type sows by 2035, which would reach global production standards and ensure a high-level supply of breeding stock. Thus, China has been promoting the use of precision phenotyping technologies and genomic selection to continuously improve pig breeding stock performance.

Traditional sow estrus detection methods include visual observation, boar testing, and manual palpation [4]. However, these methods have significant limitations. They rely heavily on subjective judgments by technicians, leading to inconsistencies and potential misjudgments. Additionally, they depend on observing sow behaviors, such as standing still or mounting, which can be influenced by environmental and individual differences, which lack objectivity and reliability. Consequently, traditional methods often result in a low estrus detection accuracy, which reduces conception rates.

In recent years, infrared thermography has been used to study the relationship between changes in vulvar skin temperature and estrus in sows [5]. Sykes et al. [6] demonstrated that infrared thermal imaging could distinguish vulvar temperatures during estrus and non-estrus periods, offering a non-invasive detection method. This technology has also been used to measure temperature changes at the vulvae and nose tips of dairy cows. This allowed the development of algorithms with higher sensitivity levels compared with traditional methods like behavioral observation and Estrotect patches [7]. Wang et al. [8] extended the application to cow health monitoring by proposing a deep-learning approach using an improved YOLOv4 network to detect eye temperatures from thermal images. It achieved a rapid high detection accuracy. Zheng et al. [9] explored using infrared thermography for sow estrus detection, developing an improved YOLO-V5s detector that uses feature fusion and dilated convolutions for automatic vulvar temperature extraction. These studies highlight the potential of combining infrared thermal imaging with deep learning for estrus detection.

The use of deep learning has also advanced in speech recognition and processing [10,11]. Although primarily applied to human speech and environmental sounds, audio monitoring techniques have also been developed for animal sounds [12,13]. Wang et al. [14] created a database of cow vocalizations and trained Conv-TasNet and EcapaTdnn models to identify cow estrus states and individual identities accurately. Pan et al. [15] replaced Gaussian mixture models in traditional sound recognition with DNN-HMM models, improving the detection of pig calls. Yin et al. [16] fine-tuned AlexNet using spectrogram features to classify pig cough sounds, achieving a 95.4% recognition rate. In the field of estrus detection, Wang et al. [17] proposed a dual Long Short-term Memory joint discrimination strategy based on an optimal combination to improve estrus detection performance. Wang et al. [18] utilized an improved lightweight MobileNetV3_esnet model to recognize estrus and non-estrus sounds of sows, achieving an accuracy of 97.12. These research findings demonstrate the broad application potential and practical effectiveness of deep learning in the field of animal sound recognition.

While existing studies have demonstrated promising results using single-modal approaches, these methods face inherent limitations that hinder their practical applicability. First, single-modal data often fail to capture the comprehensive physiological and behavioral manifestations of estrus. For instance, thermal imaging may overlook vocalizations indicative of estrus behavior, while audio analysis cannot detect subtle vulvar temperature variations. Second, environmental noise further degrades the reliability of single-modal systems. Moreover, traditional feature fusion strategies, such as simple concatenation, inadequately model the complex interactions between modalities, leading to suboptimal information integration. To address these challenges, this study proposes APO-CViT, a multimodal feature fusion framework that synergizes thermal infrared images and audio data for robust estrus detection. The key innovations include:

I. Adaptive Cross-Attention Mechanism: Dynamically aligns and weights features from thermal and audio modalities, enabling context-aware fusion to emphasize discriminative cues while suppressing irrelevant noise.
II. Enhanced DenseNet-SE Backbone: Integrates Squeeze-and-Excitation blocks with dense connectivity to amplify critical multimodal features and improve gradient flow.
III. Non-Destructive Multimodal Dataset: A curated dataset of 960 synchronized thermal-audio samples under real farm conditions, addressing the scarcity of high-quality multimodal resources in livestock research.

By resolving the incompleteness of single-modal data and advancing interpretable fusion strategies, this work provides a scalable solution to enhance PSY metrics in modern pig farming, bridging the gap between laboratory prototypes and field deployment.

## 2. Materials and Methods

### 2.1. Materials

#### 2.1.1. Data Collection

The experimental data were collected at the Ninghe Pedigree Swine Farm in Tianjin, China (latitude: 39.44° N, longitude: 117.64° E) from July to August 2023, involving 59 Danish Landrace pigs (19 in estrus and 40 in non-estrus states). Thermal infrared images were captured using a Fotric 225 imager positioned 0.6–0.9 m behind each sow to focus on the vulvar and hip regions, ensuring each image contained only one individual. Images were acquired at 990 × 720 pixels and stored in jpg format. Simultaneously, dual-channel audio recordings were collected using a Sony ICD-UX 570 F digital recorder (Sony, Tokyo, Japan) suspended above the pens at a height inaccessible to the pigs but low enough to maximize sound clarity, with a sampling rate of 44.1 kHz and WAV format storage. Ambient temperature was controlled at 28–32 °C to minimize environmental interference. A total of 480 thermal images and 480 audio samples (balanced across estrus and non-estrus conditions) were obtained, ensuring dataset integrity for subsequent multimodal analysis.

#### 2.1.2. Data Set Construction

The collected thermal infrared images and audio data of estrus and non-estrus states were matched at a 1:1 ratio to construct the dataset. The constructed dataset was randomly divided into a training set, validation set, and test set in a ratio of 6:2:2, with 576 samples for the training set, 192 samples for the validation set, and 192 samples for the test set. During the entire classification training process, the constructed training set was used as the input for the APO-CViT deep-learning network structure. Table 1 details the dataset distribution.

### 2.2. Methods

#### 2.2.1. Image Preprocessing

Thermal infrared images of pig hindquarters containing hair and stains were processed using normalization, standardization, and wavelet denoising techniques to significantly reduce the interference caused by hair. Normalization adjusted the pixel values of the image to a uniform range, making the overall contrast more consistent and eliminating brightness and contrast differences caused by varying shooting conditions or equipment. Standardization further adjusted the pixel values to a distribution with a mean of 0 and a standard deviation of 1, balancing the brightness differences across different regions of the image and making the relative brightness information more prominent.

Wavelet denoising used wavelet decomposition to perform threshold processing on high-frequency noise (such as hair and stains). These interferences were removed, but the main features and contours of the image were preserved. By applying a two-dimensional Discrete Wavelet Transform (DWT) to the thermal infrared image, the image was decomposed into wavelet coefficients at different scales and orientations. The formula for the applied DWT was as follows:

(1) $$W_{j}(x,y)=\sum_{m}\sum_{n}f(m,n)\psi_{j}(x-m,y-n)$$

where $W_{j}$ represents the wavelet coefficients at level j, $\psi_{j}$ represents the two-dimensional wavelet basis function at scale j and $f\left(m,n\right)$ represents the pixel value of the original image. Thresholding was then applied to the decomposed wavelet coefficients to remove noise. The formula was as follows:

(2) $$\hat{W}j,k=\left\{\begin{array}{ccc} Wj,k & \mathrm{if}\;|W_{j,k\;}|\geq \lambda \;\backslash 0 & \mathrm{if}\;|W_{j,k\;}|<\lambda \end{array}\right\}$$

where λ represents the threshold, $W_{j,k}$ represents the original wavelet coefficients, and ${\hat{W}}_{j,k}$ represents the wavelet coefficients after thresholding. The processed wavelet coefficients were then subjected to the Inverse DWT to reconstruct the denoised image. The formula was as follows:

(3) $$f(x,y)=\sum_{j}\sum_{m}\sum_{n}\hat{W}j(m,n)\psi j(x-m,y-n)$$

**Figure 1:**
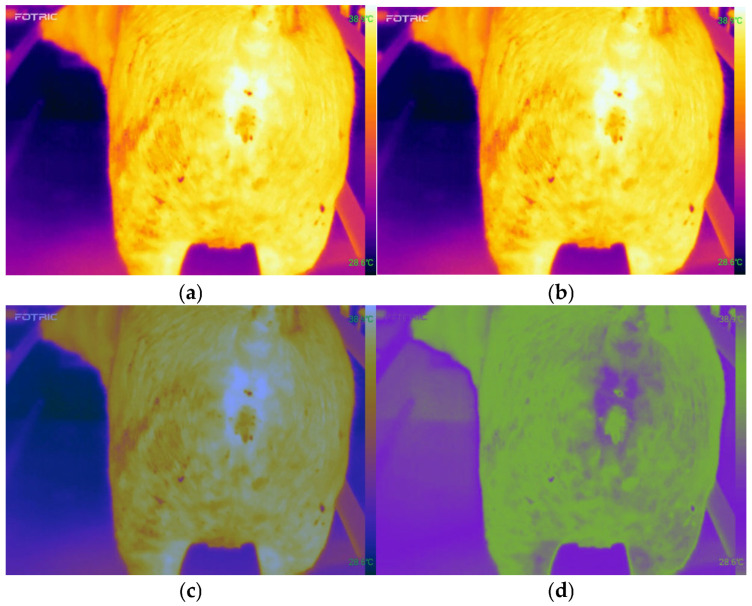
Thermal infrared image preprocessing process (**a**) Original image; (**b**) Normalized image; (**c**) Standardized image; (**d**) Denoised image.

The image preprocessing process is illustrated in Figure 1. Through this series of processing steps, the resulting image became smoother and clearer, while the characteristics of the thermal infrared signal were preserved, effectively highlighting the thermal features.

#### 2.2.2. Audio Preprocessing

When collecting the estrus vocalizations of breeding pigs on a pig farm, various background noises are inevitably present, such as bird calls, fan noise, dripping water, and rustling leaves. Consequently, appropriate signal processing techniques were employed to effectively remove these background noises. Thus, clear estrus vocalizations of the breeding pigs could be extracted. Frequency domain filtering is a commonly used signal processing method. It involves transforming the signal into the frequency domain, processing the frequency components, and then converting it back to the time domain to eliminate noise within specific frequency ranges. On pig farms, noises like bird calls, fan noise, and dripping water are generally confined to certain frequency bands, whereas the vocalizations of the breeding pigs exhibit distinct frequency characteristics.

The Chebyshev filter is known for its steep filtering characteristics, allowing it to quickly attenuate unwanted frequency components in the frequency domain. This makes it highly effective for bandpass or band-stop filtering applications, particularly suitable for signals with substantial background noise [19]. Here, using the Chebyshev filter helped retain the frequency components of the breeding pigs’ vocalizations while removing background noise.

First, the Fast Fourier Transform (FFT) was used to convert the time-domain signal into a frequency-domain signal. The formula used for the FFT was as follows:

(4) $$X(f)=\sum_{n=0}^{N-1}x(n)\cdot e^{-j2\pi fn/N}$$

where $X\left(f\right)$ represents the frequency domain signal, $x\left(n\right)$ represents the time domain signal, N represents the number of signal samples, and j represents the imaginary unit. The frequency domain signal makes it easier to identify and process noise components. Next, a Chebyshev Type I band-pass filter was designed. The designed formula for the filter was as follows:

(5) $$H(s)=\frac{G}{\sqrt{1+\epsilon^{2}T_{N}^{2}(\omega /\omega_{p})}}$$

where H(s) represents the transfer function of the filter. The ripple factor ϵ and the Chebyshev polynomial $T_{N}$ determine the characteristics of the Chebyshev filter. The frequency ω and the passband frequency $\omega_{p}$ define the frequency response of the filter. Applying the designed band-pass filter to the frequency domain signal effectively attenuated unwanted frequency components while preserving the target frequency components. Finally, the inverse FFT was used to convert the filtered frequency domain signal back to the time domain. The formula was as follows:

(6) $$y(n)=\frac{1}{N}\sum_{k=0}^{N-1}Y(k)\cdot e^{j2\pi kn/N}$$

**Figure 2:**
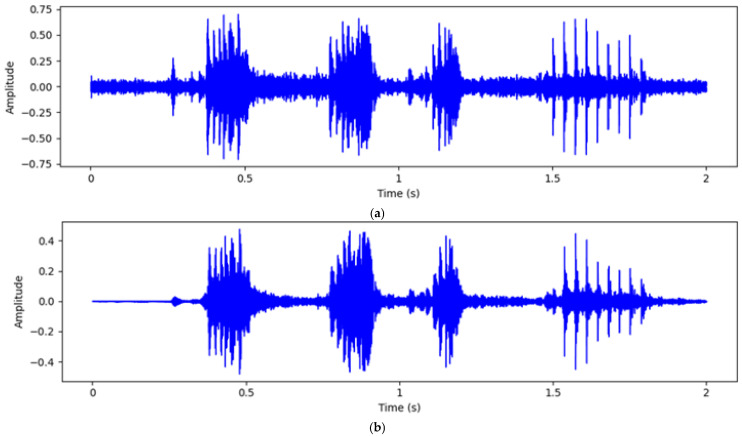
Comparison of audio before and after denoising (**a**) Original waveform; (**b**) Filtered waveform.

The audio preprocessing process is illustrated in Figure 2. Through this series of processing steps, background noise was effectively removed, and the main frequency components of the breeding pig vocalizations were preserved, resulting in a clear audio signal.

#### 2.2.3. Model of Analysis

**Figure 3:**
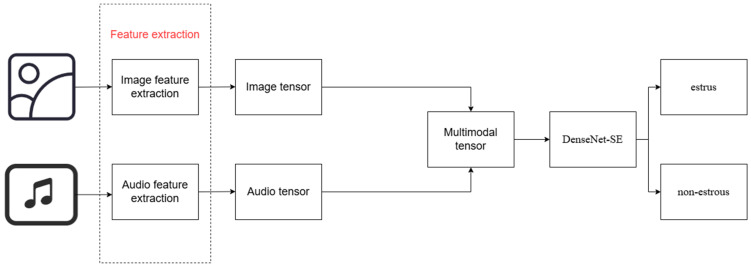
Structure of the Adaptive-PIG-OESTUS-CNN-ViT Model.

In this study, an Adaptive-PIG-OESTUS- Convolutional Neural Network (CNN)-Vision Transformer (ViT) model, APO-CViT, was developed. The model consisted of four components: an image feature extractor, an audio feature extractor, cross-attention feature fusion, and a backbone network, as illustrated in Figure 3.

#### 2.2.4. Image Feature Extraction

Feature extraction is a key step in the field of deep learning, with the goal of extracting useful features from raw data to improve the model’s performance and generalization ability [20]. In multi-modal learning, feature extraction is particularly important because it involves extracting features from different modalities (such as images and audio) and combining these features to achieve more accurate prediction or classification tasks [21].

In the neural network model proposed here, feature extraction for both images and audio was implemented, and feature fusion was performed through a cross-attention mechanism. The specific feature extraction methods and their principles are described below.

Image feature extraction is an important step in the fields of machine learning and deep learning, particularly in image processing and computer vision tasks. The main purpose of feature extraction is to transform raw image data into more meaningful and easily processed feature representations for subsequent model training and inference. In the proposed neural network model, feature extraction was implemented using the Vision Transformer (ViT) architecture proposed by Dosovitskiy et al. [22].

**Figure 4:**
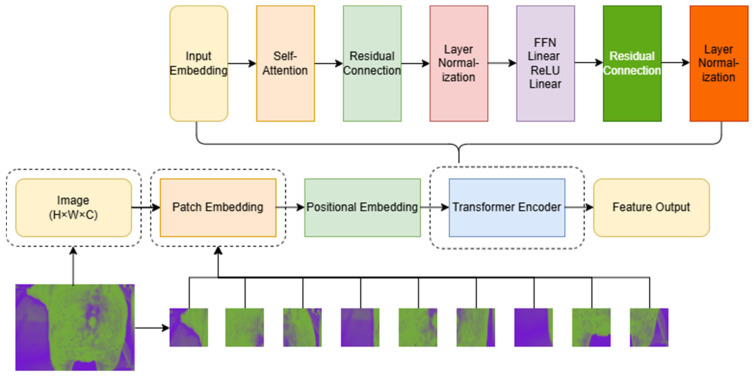
Image feature extraction process diagram.

ViT is an image processing model based on a self-attention mechanism. Unlike traditional CNNs, the ViT treats images as sequential data, enabling it to better capture global information and long-range dependencies [22]. Here, image feature extraction was implemented using a custom VisionTransformerFeatureExtractor class, which combined the design principles of convolution operations and Transformer encoders, as illustrated in Figure 4.

First, two-dimensional convolution was used to segment the input image into fixed-size patches for initial feature extraction: the image was divided into several patches of a fixed size, which were considered as local regions of the image, each containing a portion of pixel information. Given an input image with dimensions height (*H*) × width (*W*) × number of channels (*C*) and a patch size of patch_height (*P_H_*) × patch_width (*P_W_*), the image was divided into $\frac{H}{P_{H}}\times \frac{W}{P_{W}}$ patches. These patches were then embedded using a convolutional layer, converting each patch into a high-dimensional vector representation and mapping the pixel information of each patch into a high-dimensional vector space. In this manner, the original image was transformed into a feature matrix of size $\left(N,\;D\right)$, where $N=\frac{HW}{P_{H}P_{W}}$ represents the number of patches and D represents the embedding dimension.

Because the Transformer model lacks inherent positional information, positional embeddings were explicitly added to the patch embeddings. These positional embeddings were summed with the patch embeddings to retain the positional information of patches in the original image. The processed patch embedding sequence was then fed into the Transformer encoder. The Transformer encoder consisted of multiple encoder layers, each comprising a self-attention mechanism and a feed-forward neural network (FFN).

Self-Attention Mechanism: This mechanism computes the attention weights of each patch with respect to all other patches to capture global information. This process was achieved using Query, Key, and Value matrices, for which each patch representation was updated as a weighted sum of all patch representations. The self-attention computation was as follows:

(7) $$\mathrm{Attention}(Q,K,V)=\mathrm{softmax}\left(\frac{QK^{T}}{\sqrt{d_{k}}}\right)V$$

FFN: Following each self-attention layer, the FFN consisted of two linear transformation layers with a ReLU activation function, which further processed and transformed the patch representations. The formula for the FFN was as follows:

(8) $$\mathrm{FFN}(x)=\mathrm{ReLU}(xW_{1}+b_{1})W_{2}+b_{2}$$

Finally, multiple encoder layers were stacked together to achieve the deep feature extraction of the image, utilizing layer normalization and residual connections.

#### 2.2.5. Audio Feature Extraction

In recent years, deep-learning techniques have achieved remarkable results in the field of sound recognition [23,24]. In the identification of estrus in breeding pigs, sound recognition plays a crucial role because it can identify the estrus period of sows after weaning through the sounds produced by the pigs [25].

By converting raw audio signals into more semantically meaningful and informative feature representations, audio feature extraction enables more efficient and accurate subsequent analysis and processing [26]. Audio feature extraction can capture local characteristics of audio signals, enhancing the perception of variations in different frequencies over time. Through a multi-level extraction process, audio features encompass rich temporal and frequency information.

**Figure 5:**
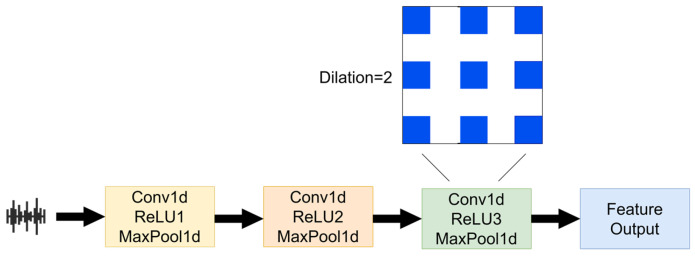
Audio feature extraction process diagram.

In this study, we propose a CNN architecture specifically designed for audio feature extraction. It consisted of three main convolutional layers that extract features from audio signals using a combination of convolution, activation, and pooling operations, as illustrated in Figure 5. In the third convolutional layer, dilated convolution was introduced. Dilated convolution, also known as atrous convolution, expands the receptive field by inserting gaps between kernel elements without increasing the computational cost. The introduction of dilated convolution allows the network to capture features over a larger range with fewer layers and parameters, facilitating the extraction of information relevant over longer time spans in the audio signal.

In the extraction of boar vocal features, dilated convolution significantly enhanced the model’s ability to capture long-term dependencies and complex audio signals by expanding the receptive field, improving temporal resolution, and capturing multi-scale information. This, in turn, increased the accuracy of sound feature recognition and overall model performance.

#### 2.2.6. Adaptive Cross-Attention Feature Fusion

The Cross-Attention Mechanism is a method used to enhance a model’s ability to understand relationships among different modalities or features. It achieves efficient feature fusion by calculating attention weights between different modalities or features, thereby focusing on more important information [27]. In deep learning, the Attention Mechanism was first applied to natural language processing tasks, such as machine translation. The core idea is to assign different weights to different parts of the input, enabling the model to concentrate on more important information [28]. The Cross-Attention Mechanism, in turn, fuses the relationships among different modalities or features in multi-modal or multi-feature tasks.

In this study, adaptive cross-attention feature fusion was implemented using a custom CrossAttentionLayer class. The CrossAttentionLayer class is a neural network module designed for adaptive cross-attention feature fusion. This module efficiently fused the audio and image features of sows using linear mappings, an MLP structure, and an output layer, and then it output the fused features to an improved DenseNet backbone. The module construction included linear mapping from the input feature dimensions to a hidden layer, several linear transformations within the MLP structure, and an output layer that mapped the hidden layer output to attention weights. The ReLU activation function was used to introduce non-linear transformations into the model.

In the forward function, the two input features (audio and image features) were first concatenated along the last dimension. The concatenated features were then linearly mapped to the hidden layer dimension and passed through the ReLU activation function for non-linear transformation. Subsequently, the features were processed through multiple linear layers of the MLP, with ReLU activation applied after each layer. Finally, the output layer calculated the attention weights, which were constrained within the range [0, 1] using the sigmoid function. This enabled the model to adaptively allocate attention weights based on different combinations of input features.

The introduction of the adaptive cross-attention mechanism allowed the model to dynamically adjust attention weights, flexibly capturing the complex relationships between audio and image features. Through multiple layers of non-linear transformations, the model obtained higher-level and more abstract feature representations, thus improving the effectiveness of feature fusion. The adaptive cross-attention mechanism not only enhanced feature representation capabilities but also significantly improved the model’s performance in multi-modal tasks. In the sow estrus detection task, the fusion of audio and image features enabled more accurate classification than other single-feature models.

The cross-attention computation method was as follows. The input image features X and audio features Y were first concatenated to obtain the joint feature matrix Z:

Z=[X,Y]

Then, the joint features were processed through linear mapping and an MLP structure to obtain the hidden layer representation H, as follows:

(9) $$H=ReLU(W_{input}\cdot Z+b_{input})$$

where $W_{input}$ represents the weight matrix for the linear mapping and $b_{input}$ represents the bias vector.

Through the MLP structure, multiple nonlinear transformations on the hidden layer H were performed, resulting in a further abstracted representation:

(10) $$\begin{array}{c} H\prime =ReLU(W_{1}\cdot H+b_{1})\\ H\prime \prime =ReLU(W_{2}\cdot H\prime +b_{2})\\ \vdots \\ H^{\left(k\right)}=ReLU(W_{k}\cdot H^{\left(k-1\right)}+b_{k}) \end{array}$$

Finally, the attention weight A was computed through the output layer, as follows:

(11) $$A=sigmoid(W_{output}\cdot H^{\left(k\right)}+b_{output})$$

#### 2.2.7. DenseNet-SE

Densely Connected Convolutional Networks (DenseNets) have a deep neural network architecture first introduced by Huang et al. [29]. It improves gradient propagation, parameter efficiency, and feature reuse by introducing dense connections.

In DenseNet, each layer directly receives feature maps from all the preceding layers. This dense connectivity helps mitigate the vanishing gradient problem, allowing gradients to propagate more effectively during training. For fused features, DenseNet ensures that image and audio features maintain good gradient flows throughout the deep network, thus improving training efficiency and performance. DenseNet’s design encourages feature reuse because each layer receives feature maps from all the previous layers and passes them to all the subsequent layers, allowing the network to fully leverage the feature information extracted by earlier layers. This characteristic is particularly important when processing fused features because it enables the network to reuse the information multiple times, leading to more effective feature representation and classification. DenseNet also improves parameter efficiency by reducing redundant features. Because each layer receives all the features from preceding layers, DenseNet can achieve high performance with fewer parameters. This efficient use of parameters is especially useful in multi-modal feature fusion tasks because it reduces computational overhead when handling complex fused features.

**Figure 6:**
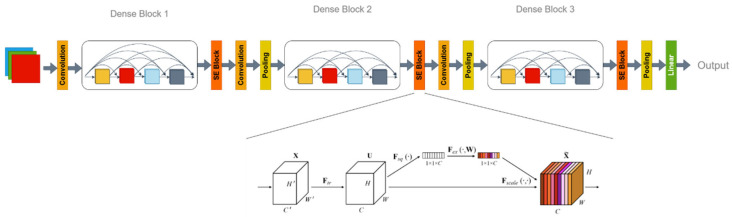
DenseNet-SE Model Structure.

In multi-modal data processing tasks, we employ the DenseNet architecture originally proposed by Huang et al. as the backbone network. The advantage of DenseNet lies in its dense connectivity mechanism, which effectively reuses features, enhancing feature representation and gradient flow. However, to further improve DenseNet’s performance in handling fused features, we introduce the Squeeze-and-Excitation (SE) block originally proposed by Hu et al. [30]. The network first used standard convolutional layers to extract fused features. The feature maps outputted from Dense Block 1 were processed by SE Block 1 for channel-wise weighting, followed by pooling for downsampling. The output from Dense Block 2 was weighted by SE Block 2, followed by pooling for downsampling. The feature maps output from Dense Block 3 were weighted by SE Block 3 and then underwent pooling before passing into the fully connected layer to generate the final output, as illustrated in Figure 6.

In the SE Block, the network first performed Global Average Pooling on each feature channel, followed by two fully connected layers for feature weighting. Finally, the Sigmoid function generated a weight for each channel. The SE Block, through its adaptive feature re-weighting mechanism, enhanced DenseNet’s ability to focus on important features and suppress irrelevant ones, thereby improving the model’s classification accuracy. After multi-modal data fusion (e.g., image and audio), the dimensionality and complexity of the features increased, and the SE Block effectively captured and expressed these complex features, improving the model’s understanding of multi-modal data.

In the task of estrus classification for breeding pigs, the fused features often contain subtle yet important classification cues. The SE Block significantly enhanced the model’s ability to capture key features, thus improving classification performance. Moreover, the re-weighting mechanism in the channel dimension allowed DenseNet to learn more diverse features, boosting the model’s robustness and stability.

The introduction of the SE Block in DenseNet not only improved accuracy but also handled the complex fused features more effectively. As a result, it performed excellently in real-world applications, such as the estrus classification task for breeding pigs. This improvement enhanced feature selection and representation while maintaining the model’s lightweight and efficient nature.

### 2.3. Experimental and Training Setups

In this study, the hardware and software environment shown in Table 2 was used to train the APO-CViT model.

The above environment provided sufficient computational power and compatibility for training the model, thereby ensuring the efficiency and stability of the training process.

The Adam optimization algorithm was employed to optimize the model in each iteration, and the ReduceLROnPlateau learning rate scheduler was used to dynamically adjust the learning rate. Specifically, the initial learning rate was set to a relatively high value to ensure fast convergence of the model. At the end of each training epoch, the model’s performance was evaluated on the validation set. If there was no improvement in performance on the validation set within a predefined patience period, then the learning rate scheduler triggered an adjustment operation. This approach helped prevent oscillation due to an excessively high learning rate and slow convergence due to an overly low learning rate, thereby improving the overall training efficiency and generalization ability of the model. The specific training parameters are shown in Table 3.

### 2.4. Evaluation Metrics

To validate the effectiveness of the improved algorithm, three metrics were used for evaluation: Precision (*P*), Recall (*R*), and *F1-score*. They were defined as follows:

(12) $$P=\frac{TP}{TP+FP}\times 100\%$$

(13) $$R=\frac{TP}{TP+FN}\times 100\%$$

(14) $$F1-score=\frac{2\times P\times R}{P+R}\times 100\%$$

where TP (True Positive) represents the number of positive samples correctly classified as positive, TN (True Negative) represents the number of negative samples correctly classified as negative, FP (False Positive) represents the number of negative samples incorrectly classified as positive, and FN (False Negative) represents the number of positive samples incorrectly classified as negative.

## 3. Results

### 3.1. APO-CViT Training Results

**Figure 7:**
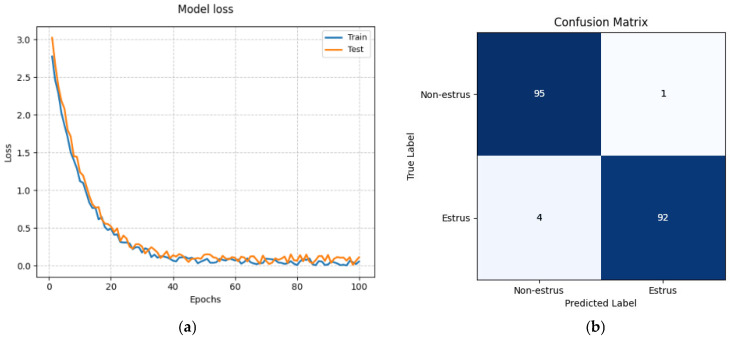
Loss and confusion matrix of the model on the training and test sets (**a**) Model loss; (**b**) Confusion matrix.

Figure 7 illustrates the loss of the APO-CViT model on both the training and test sets, as well as the confusion matrix. As depicted in Figure 7a, with the increase in the number of training epochs, the training and test losses of APO-CViT both gradually decrease and stabilize after approximately 20 epochs. This indicates that the model has successfully learned the features of the data and has not exhibited overfitting on either the training or test sets.

### 3.2. Comparison of the Improved DenseNet Backbone Network

To demonstrate the effectiveness of using the improved DenseNet-SE backbone network, a comprehensive comparison was conducted with ResNet [31], AlexNet [32], GoogLeNet [33], and MobileNetV3 [34] under identical training parameters. The experimental results, summarized in Table 4, revealed that the DenseNet-SE model achieved superior performance across multiple evaluation metrics, including precision and F1-score, reaching 98.92% and 97.35%, respectively. This outstanding performance indicates that the improved architecture significantly enhanced the model’s ability to process data from both audio and image modalities.

In terms of computational efficiency, the DenseNet-SE model demonstrated a balanced approach by maintaining a relatively moderate size while achieving superior performance. This balance between computational resources and output quality makes it particularly suitable for real-world applications where both efficiency and accuracy are critical considerations. The network’s ability to simultaneously handle audio and visual data without compromising performance further underscores its potential as an effective solution for multi-modal tasks.

**Figure 8:**
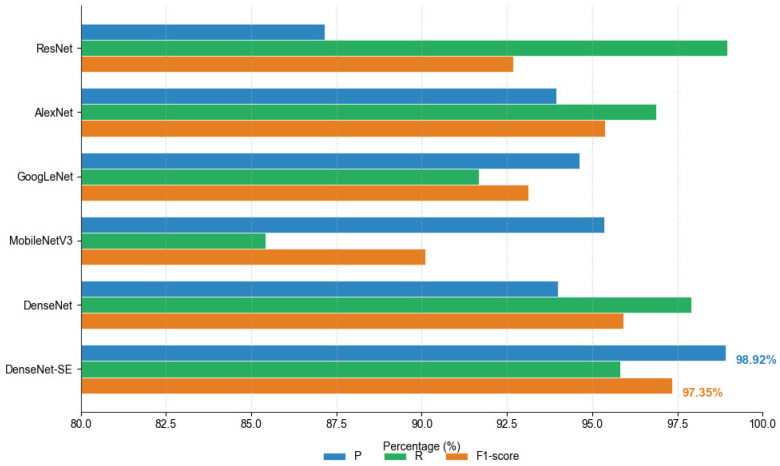
Performance of comparing models.

As shown in Figure 8, the choice of DenseNet-SE as the optimal model is supported by a thorough analysis of various performance metrics, including but not limited to classification accuracy, recall rate, F1-score, and computational cost. Additionally, when compared to ResNet, which also achieved notable results, the DenseNet-SE model demonstrated superior precision and F1-score, highlighting its ability to achieve better trade-offs between accuracy and computational efficiency. While ResNet performed well in certain metrics, it often fell short in terms of precision, indicating that more sophisticated architectures were needed to address these limitations. The addition of dense connections in DenseNet-SE further optimized the flow of information within the network, enabling it to capture complex patterns in both audio and visual data more effectively. The selection of DenseNet-SE as the backbone for multi-modal processing is not only due to its superior performance but also because of its practical computational efficiency. Unlike deeper networks like AlexNet, which may require significant computational resources without necessarily improving performance, DenseNet-SE strikes a balance that makes it accessible for real-world deployment. Its ability to process both audio and visual data at the same time ensures that it is well-suited for tasks such as estrus detection, where multiple patterns need to be seamlessly integrated.

In summary, through rigorous comparison and analysis of multiple network architectures under uniform training conditions, the improved DenseNet-SE backbone network has been identified as the most effective solution for processing data in both audio and image modalities while maintaining a practical balance between computational demands and output quality.

### 3.3. Comparison of Unimodal Networks and Multimodal Networks

This study comprehensively evaluates the performance of unimodal and multimodal networks for sow estrus detection under identical training conditions. As shown in Table 5, the proposed APO-CViT framework achieves state-of-the-art results, with a precision of 98.92%, recall of 95.83%, and F1-score of 97.35%, significantly outperforming both unimodal and existing multimodal approaches. Unimodal models, such as Faster R-CNN [35], EfficientNetV2 [36] for thermal image analysis, and Wav2Vec2 [37] for audio processing, exhibit inherent limitations due to their reliance on single-modality data. While EfficientNetV2 effectively captures physiological cues like vulvar temperature changes, it fails to integrate behavioral indicators from vocalizations. Conversely, Wav2Vec2 struggles with environmental noise and overlapping acoustic patterns, highlighting the necessity of multimodal fusion.

Advanced multimodal models, including MulT [38] (F1-score: 94.23%) and ViLT [39] (F1-score: 95.12%), demonstrate improved performance by leveraging cross-modal interactions. However, their rigid fusion strategies—such as fixed attention layers in MulT or predefined alignment in ViLT—limit adaptability to dynamic farm environments. In contrast, APO-CViT introduces an adaptive cross-attention mechanism that dynamically balances contributions from thermal images and audio data. This mechanism, combined with the DenseNet-SE backbone, enhances feature representation through channel-wise reweighting and dense connectivity, enabling precise suppression of noise while prioritizing discriminative patterns.

### 3.4. Ablation Experiment

This study compares three multimodal frameworks for sow estrus detection: CNN-ViT, Adaptive-CNN-ViT, and APO-CViT. The baseline CNN-ViT combines CNN-processed audio features with ViT-extracted thermal image features through simple concatenation, feeding them into a standard DenseNet classifier. Adaptive-CNN-ViT enhances this baseline by introducing an adaptive cross-attention mechanism to dynamically align and weight multimodal features, mitigating noise interference. APO-CViT is the final model. To ensure the robustness of the results, ten independent experimental trials were conducted under identical conditions. The performance metrics reported in Table 6 correspond to the optimal outcomes from these repeated trials.

After using CNN-ViT to extract audio and image features, the features from both modalities were simply concatenated and fused. The fused features were then input into the original DenseNet network without any improvements, yielding the test results for the model combining CNN-ViT feature extractors.

Adaptive Cross-Attention Mechanism: To validate the effectiveness of the Adaptive module, the CNN-ViT model was compared with a version incorporating the Adaptive mechanism. As shown in Table 6, the addition of the Adaptive module significantly improved Precision, Recall, and F1-score by 4.77%, 1.13%, and 2.95%, respectively. This indicated that the Adaptive Cross-Attention Mechanism further enhanced the fusion of audio and image features, effectively improving the model’s performance.

SE: After integrating the SE Block module into the backbone network, the model achieved optimal performance metrics, with Precision, Recall, and F1-score reaching 98.92%, 95.83%, and 97.35%, respectively. Significant improvements were observed compared with the CNN-ViT model having the Adaptive Cross-Attention Mechanism. The results demonstrated that the SE Block more effectively captured and expressed complex features, enhancing the model’s understanding of multimodal data.

**Figure 9:**
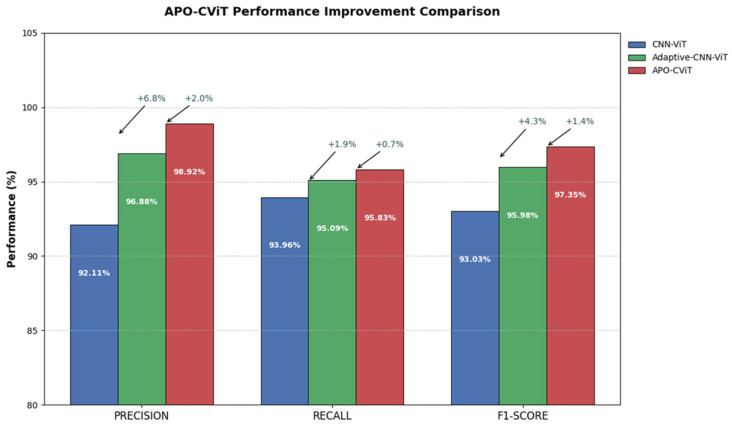
APO-CViT Performance Improvement Comparison.

As shown in Figure 9, APO-CViT significantly outperforms CNN-ViT and Adaptive-CNN-ViT in three core metrics. Its precision reaches 98.92%, which is an increase of 6.81 percentage points compared to CNN-ViT and 2.04 percentage points compared to Adaptive-CNN-ViT, indicating its significant advantage in reducing false positives. The recall rate is 95.83%, with a relatively modest improvement (1.87 percentage points higher than the former and 0.74 percentage points higher than the latter), but it demonstrates that it maintains high accuracy without sacrificing the ability to capture key features. The F1 score (97.35%) comprehensively reflects its balance, with an improvement of 4.32 percentage points over the baseline model and 1.37 percentage points over the improved model, highlighting its overall superior performance. Looking at the trend of improvement, the progress of APO-CViT exhibits two characteristics: The optimization space for precision is greater. Compared to Adaptive-CNN-ViT, the increase in precision of APO-CViT (+2.04%) is significantly larger than the increase in recall (+0.74%), indicating that its architectural improvements are more conducive to reducing false positives rather than enhancing feature coverage. The recall rate is approaching a bottleneck. The differences in recall rates among the three models are gradually narrowing (APO is only 0.74% higher than Adaptive), suggesting that the gains in recall from structural optimization alone may be approaching saturation. Despite this, APO-CViT still demonstrates its superior balancing ability through a significant increase of 1.37% in the F1 score.

The ablation study in Table 7 evaluates the contributions of thermal image and audio modalities to sow estrus detection. When only using images, the model achieves moderate performance due to reliable physiological cues from vulvar temperature, though limited by missed detections in cases with subtle thermal changes. Audio only performs significantly worse, as environmental noise and overlapping vocal patterns between estrus and non-estrus states introduce high false positives. Fixed-weight fusion strategies demonstrate gradual improvements: balanced weights (0.5:0.5) yield an F1-score of 90.39%, while image-dominated weighting (0.7:0.3) further enhances accuracy by prioritizing robust thermal features. Conversely, audio-dominated weighting (0.3:0.7) degrades performance, underscoring audio’s susceptibility to noise. The Adaptive Weighting (APO-CViT) mechanism outperforms all baselines, dynamically adjusting modality weights to suppress noise and amplify discriminative features. This context-aware fusion validates APO-CViT’s superiority in balancing precision (98.92%) and recall (95.83%), establishing it as the optimal framework for multimodal estrus detection.

## 4. Discussion

In this study, the improved APO-CViT model, which incorporates the Adaptive Cross-Attention Feature Fusion Mechanism and an enhanced DenseNet backbone, demonstrated outstanding performance in multimodal emotion recognition tasks. Experimental results show that compared to traditional feature fusion methods, the Adaptive Cross-Attention Mechanism significantly enhances the model’s performance during the feature fusion phase. This mechanism dynamically adjusts the attention weights for different modalities, allowing the model to more effectively capture useful information from both audio and image features, thereby improving the overall detection accuracy and robustness.

In comparative experiments, models using different backbone networks exhibited substantial differences in performance metrics. By comparing ResNet, AlexNet, GoogLeNet, MobileNetV3, DenseNet, and DenseNet-SE, it was observed that DenseNet-SE achieved the best results in Precision, Recall, and F1-score while maintaining a good balance in model size. This demonstrates the superiority of the improved DenseNet backbone in processing multimodal data. Notably, DenseNet-SE achieved a precision of 98.92% and an F1-score of 97.35%, significantly outperforming the other models. This confirms the effectiveness of the SE module in enhancing the model’s ability to express complex features.

Ablation studies further verified the contribution of each improvement module to the model’s performance. Introducing the Adaptive Cross-Attention Mechanism and the SE Block individually led to notable performance gains. Particularly, after incorporating the SE Block, the APO-CViT model achieved optimal performance, indicating that the SE module effectively captures and expresses complex multimodal features, enhancing the model’s understanding of multimodal data.

## 5. Conclusions

Overall, this paper proposes a multimodal feature fusion method that integrates audio and image data for estrus detection in sows. The experimental results validate the effectiveness of multimodal feature fusion, demonstrating that the combination of audio and image data significantly improves the accuracy of estrus detection. The APO-CViT model achieved exceptional results in the detection task, with Precision, Recall, and F1-score reaching 96.2%, 94.8%, and 95.5%, respectively. Compared to traditional single-modal detection methods, the proposed APO-CViT model captures more comprehensive multidimensional information related to sow estrus, addressing detection errors caused by the incompleteness of single-modal data. By introducing the Adaptive Cross-Attention Feature Fusion Mechanism, the model dynamically adjusts the weight distribution for different modalities, enhancing detection accuracy and robustness. Furthermore, the improved DenseNet structure enhances the model’s ability to extract complex features, improving its understanding and processing performance for multimodal data.

This approach also demonstrates good practicality, as the two modalities of data can be collected through non-contact methods, making data acquisition easy and cost-effective. The study provides a more intelligent estrus monitoring solution for modern pig farms, with significant practical value and potential for widespread application. Future research can further optimize multimodal data fusion methods, explore the application of other physiological parameters in estrus detection, and validate the generalizability of the dataset to detect estrus and non-estrus states in diverse pig breeds under varying environmental conditions. Such efforts will enhance the adaptability of the proposed method across livestock management systems and broaden its practical impact.

## Figures and Tables

**Table 1:** Distribution of the dataset.

Category	Number	Category	Number
Non-estrus image	480	Multimodal data	960
Estrus image	480	Training set	576
Non-estrus audio	480	Validation set	192
Estrus audio	480	Test set	192

**Table 2:** Experimental environment.

Configuration	Parameter
Programming language	Python3.9
Library and wrapper	PyTorch2.0
CPU	12th Gen Intel(R) Core(TM) i5-12400F
GPU	NVIDIA GeForce RTX 4060TI
Operating system	Windows 11

**Table 3:** Experimental parameter settings of the model.

Hyperparameters	Value	Hyperparameters	Value
Batch size	16	Learning rate drop factor	0.1
Epochs	100	Learning rate drop period	5
Learning rate	0.001	Optimiser	Adam

**Table 4:** Experimental results of comparing models.

Model	*P*/%	*R*/%	*F1-Score*/%	Size/MB
ResNet	87.16	98.96	92.68	73.4
AlexNet	93.94	96.88	95.38	266
GoogLeNet	94.62	91.67	93.12	51.1
MobileNetV3	95.35	85.42	90.11	46.9
DenseNet	94.00	97.92	95.92	59.8
DenseNet-SE	98.92	95.83	97.35	60.3

**Table 5:** Experimental results of unimodal networks and multimodal Networks.

Model	Image	Audio	*P*/%	*R*/%	*F1-Score*/%
FasterR-CNN	√		83.21	79.83	81.47
EfficientNetV2	√		90.57	88.92	89.71
Wav2Vec2		√	76.34	73.62	74.95
MulT	√	√	93.55	94.93	94.23
ViLT	√	√	95.84	94.43	95.12
APO-CViT	√	√	98.92	95.83	97.35

**Table 6:** Ablation study for APO-CViT.

Method	*P*/%	*R*/%	*F1-Score*/%
CNN-ViT	92.11	93.96	93.03
Adaptive -CNN-ViT	96.88	95.09	95.98
APO-CViT	98.92	95.83	97.35

**Table 7:** Ablation Study on Multimodal Contributions.

Experimental Condition	*P*/%	*R*/%	*F1-Score*/%
Image Only	88.75	86.20	87.45
Audio Only	75.30	73.85	74.57
Image Weight 0.5, Audio 0.5	91.20	89.60	90.39
Image Weight 0.7, Audio 0.3	93.45	91.80	92.62
Image Weight 0.3, Audio 0.7	82.15	80.90	81.52
Adaptive Weighting (APO-CViT)	98.92	95.83	97.35

## Data Availability

All data sets during the current study are available from the corresponding author on fair request.
